# The mediating role of psychological well‐being on the effect of fear of future violent events at work on nurses’ intention to migration

**DOI:** 10.1111/inr.13001

**Published:** 2024-07-12

**Authors:** Deniz Güneş, Nevin Günaydin, Mustafa Amarat

**Affiliations:** ^1^ Department of Healthcare Management, Faculty of Health Sciences Ordu University Ordu Turkey; ^2^ Department of Nursing, Faculty of Health Sciences Ordu University Ordu Turkey

**Keywords:** AMOS, fear of violence, HAYES, intention to migrate, mediating role, nurse, psychological well‐being, quantitative research, violence in hospital

## Abstract

**Aim:**

To evaluate the effect of the fear of violence of nurses working in Turkey on their intention to migrate and to examine the mediating role of psychological well‐being on this effect.

**Background:**

Many countries are concerned about nurses’ fear of violence and their intention to migrate. The fear of violence at work may influence nurses’ intention to migrate. Additionally, psychological well‐being may impact this process. However, the impact of the fear of violence at work on the intention to migrate, as well as the moderating effect of psychological well‐being in a nursing context, is not well understood.

**Methods:**

We conducted this cross‐sectional study on a sample of 221 nurses from two public hospitals. We collected survey data between November 2022 and January 2023 using three scales. We analyzed the data using SPSS, AMOS, and HAYES. We followed the STROBE statement guidelines for cross‐sectional studies.

**Results:**

Nurses reported a moderate intention to migrate and a moderate to high level of fear of violence and psychological well‐being. Fear of violence is positively associated with the intention to migrate. Mediation analyses indicated that the association between fear of violence and intention to migration was mediated by psychological well‐being.

**Discussion and conclusion:**

The fear among nurses of being exposed to violence increases their intention to migrate. However, high psychological well‐being can reduce this intention.

**Implications for nursing and health policy:**

Nursing managers, policymakers, and decision‐makers need to take serious precautions against the fear of violence in the future and make necessary improvements for nurses who witness violence. To achieve this, it can begin by paying attention to the high psychological well‐being of each nurse.

## INTRODUCTION

The number of nurses per patient and their distribution within a country is essential for countries to reach their health goals (WHO, 2020a). Increasing the number of nurses per patient may be possible through national resources and employing nurses from other countries. Globally, there needs to be more skilled health workers. The severity of this issue may differ between low‐, middle‐, and high‐income nations (Aluttis et al., 2014). Many high‐income countries pursue policies to migrate foreign‐trained, skilled nurses, thereby increasing the number of workers in destination countries (Yakubu et al., 2022). The World Health Organization has reported a 60% increase in migrant nurses and doctors working in higher‐income target countries over the past decade (WHO, 2020b). In certain nations, migrant nurses constitute 20% of the total nursing workforce (Goh & Lopez, 2016). However, the policies of high‐income countries have a negative impact on the healthcare systems of source countries. The migration of nurses detrimentally impacts the accessibility, effectiveness, and efficiency of healthcare services (Top et al., 2010).

## BACKGROUND

Violence in the health sector against health professionals refers to verbal abuse, physical assaults, harassment, bullying, intimidation, threatening, and discrimination (Escribano et al., 2019) and is defined as “any act or threat of verbal or physical violence, harassment, intimidation, or other threatening disruptive behavior that occurs at the worksite with the intention of abusing or injuring the target” (Al‐Qadi, 2021). It is a serious concern in the worldwide healthcare industry. Research shows that workplace violence against health workers has a high prevalence rate. According to an umbrella review conducted by Rossi et al. (2023) the prevalence of violence in healthcare workers’ workplaces ranges between 34.1% and 78.9%. In Turkey, research suggests that the rate of violence against healthcare workers ranges between 44% and 88% (Çam & Üstüner Top, 2022; Dursun & Aytaç, 2021; Er et al., 2021; Hamzaoglu & Türk, 2019; Pınar et al., 2017). The prevalence of exposure to workplace violence among healthcare workers varies depending on the nation, working unit, and occupational category (Babiarczyk et al., 2020; Rossi et al., 2023), however, nurses are reported as the most affected occupational group experiencing violence. For example, in a study conducted in Switzerland, nurses were discovered to be by far the most afflicted professional category by all forms of violence relative to physicians, academics, medical‐therapeutic and medical‐technical workers, and administrative and other service people (Stahl‐Gugger & Hämmig, 2022). A study conducted in Turkey reported that although 66% of nurses experience violence in the workplace, 64.2% of research assistant doctors, 63.6% of specialist doctors, 41% of other healthcare workers (such as health officers, technicians, biologists, dietitians, chemists, physiotherapists), and 39.4% of technicians are exposed to workplace violence (Er et al., 2021). Increased violence in health care may cause transient or long‐lasting fear of violence in those who have been exposed to violence, those who have observed it, and those who have never seen it (Sim et al., 2020). Thus, it is believed that fear of violence may also impact the intention to migrate.

Turkey is a crucial source country for training health workers who migrate to other countries. However, the migration of trained health professionals may cause some short term and long term issues in the Turkish health system. Considering the data from the Ministry of Health (2023), in Turkey, there are 343 nurses per 100,000 patients, whereas the OECD and EU average stands at 942 and 873, respectively (Switzerland, 1871; Norway, 1856; Iceland, 1635). These figures illustrate that Turkey has a lower ratio of nurses per patient compared with OECD and European countries. Consequently, nurses in Turkey are required to perform exceptionally. In order to ensure the continuity of health services in countries with a low nurse‐to‐patient ratio, it is important to follow policies to retain existing nurses and to establish educational institutions and organizations that will increase the number of nurses.

The health sector and the nursing profession are partially in a grave crisis, especially with many nurses immigrating abroad to search for better working conditions, and this situation harms public health (Dywili et&amp;#x000A0;al., 2013). The reasons for the intention to migrate include personal characteristics (Fouarge et al., 2019), lack of job and career opportunities (Thapa & Shrestha, 2017), low salary, poor working conditions (Kadel & Bhandari, 2019), and burnout (Havaei, 2021), which seem to be influential in the formation of nurses’ intention to migrate. Furthermore, workplace violence may increase the intention to migrate (Boafo, 2016).

Recent studies show that in many countries, including Turkey, the prevalence of workplace violence experienced by nurses is high. The migration of nurses can negatively impact people's access to health services and the quality of healthcare services in the source country (Okafor & Chimereze, 2020). The intensity of violence against nurses may cause temporary or permanent fear of violence in those who have been exposed to violence, those who have witnessed it, and those who have never seen it (Sim et al., 2020). Thus, it is believed that fear of violence may also impact the intention to migrate.

H 1 Fear of violence increases the intention to migrate.

Psychological well‐being (PWB) is described in order to manage the existential challenges people face in their lives, such as continuing meaningful aims, personal development, and building good quality relations with others (Keyes et al., 2002). The literature initially described PWB as experiencing positive emotions more than negative ones (Bradburn, 1969). When the previous studies on violence, witnessing, and fear of violence are examined, it is associated with burnout, dissatisfaction (Liu et al., 2019b), psychological stress (Havaei & Macphee, 2021), and mental health (Havaei, 2021) on nurses’ psychiatry. Given these findings, it is assumed that nurses' fear of being exposed to violence will negatively affect their PWB. Therefore, hypothesis H2 was developed.

H 2 Fear of violence adversely affects PWB.

The research used the concept of “PWB” as a mediator variable. Current studies concentrate on the psychological health of nurses (Li & Hasson, 2020). Healthy and productive nurses can cope with the difficulties of the nursing profession, provide exemplary patient care, and contribute to the nursing profession (Ratanasiripong & Wang, 2011). Thus, the better the PWB of nurses, the more effective and successful they will be in their academic and clinical training. Nurses' fear of violence may affect their PWB, which may negatively affect the institution where they work and their country of origin, such as their turnover intention and migration intention. Therefore, hypothesis H3 was developed.

H 3 PWB plays a mediating role in the effect of fear of violence on the intention to migrate.

## RESEARCH AIM AND OBJECTIVES

This study aims to ascertain the effect of nurses’ fear of being exposed to violence on their intention to migrate and the mediating effect of PWB in this effect.

## METHODS

### Design, setting, and participants

This a cross‐sectional research study, was carried out between November 2022 and January 2023. The STROBE statement checklist for cross‐sectional research was applied to report this study. Participants meeting the following inclusion criteria were included: (1) actively practicing nurses, (2) with at least 1 year of experience in nursing, (3) familiar with the study's purpose, and (4) volunteering to participate. The opinions of eligible nurses were collected using a hybrid (online and face‐to‐face) method. The in‐person questionnaires were completed by a researcher holding a doctorate in psychiatric nursing. During these interviews, participants received a thorough written and verbal briefing on the study's aims. In contrast, online participants were exclusively presented with the research's goal and relevance through written methods. The reason for conducting an online survey is that nurses on annual leave or working in intensive care units could not be reached. The study was conducted in public hospitals in Ordu province. All working nurses (*N* = 723) who met the inclusion criteria were accepted as the study population. Nurses were recruited using the convenience sampling method. In the in‐person survey interviews, the participants' responses were collected in sealed envelopes, and anonymity of the procedure was maintained. In addition, participants had the right to withdraw from this study at any time and for any reason.

The sample size was determined using the G*Power 3.1 tool through power analysis. Because no study in the literature used the three scales together, the effect size was determined to be 0.2, which is the lowest effect size, using Cohen's coefficient. With an 80% power and a 95% confidence interval, the initial sample size was determined as 199 people. To account for potential design effects arising from nonnormal data distribution, the sample size was increased by 10%. Eventually, the final sample included 221 nurses.

### Instruments

#### General information

The first step of the questionnaire includes socioeconomic details such as the participant's age (in years), gender, marital status, educational background, and current duties. Lastly, the participants were asked about the duration of their tenure in the organization.

#### Fear of violence

Fear of violence was measured using the Fear of Future Violent Events at Work (FFVEW). Rogers (1994) created the instrument, and a Turkish validity and reliability assessment of the scale was conducted by Akbolat et al. (2021). The FFVEW scale comprises statements addressing both physical (e.g., hitting, kicking, grabbing, shoving, biting) and nonphysical violence (e.g., threats involving weapons, verbal abuse) that participants may experience or fear of being exposed to in the workplace next year. The scale consists of 10 items. A sample item is “I am afraid of being a victim of workplace violence.” Each item is rated on a five‐point Likert scale, ranging from one (strongly disagree) to five (strongly agree). The total score is the average of all items, with higher scores indicating greater fear of future workplace violence. The Cronbach's alpha value of the scale was 0.94 in both the original and the Turkish validity versions, whereas in this study, it is 0.96. Moreover, confirmatory factor analyses (CFA) were performed. The findings indicated that chi‐square (CMIN: 61.572), degrees of freedom (DF: 29), CMIN/DF (2.123), goodness‐of‐fit index (GFI: 0.951), normed fit index (NF: 0.976), comparative fit index (CFI: 0.987), adjusted goodness‐of‐fit index (AGFI: 0.907), and root mean square error of approximation (RMSEA: 0.071) all supported (Meydan & Şeşen, 2015) the overall measurement quality.

#### Intention to migration

Many valid and reliable tools exist in the literature on the intention to migrate. However, the Attitude Scale for Brain Drain (ASBD) developed by Öncü et al. (2018) was used because of the scale's specific focus on assessing attitudes toward migration, particularly within research exploring factors influencing migration intention among nurses and nursing students. The scale has 16 statements addressing push and pull variables (such as professional opportunities, freedom of thought, and bad experiences). A sample item is “I think that working abroad will increase my living standards.” Every item is rated on a five‐point Likert scale, ranging from one (strongly disagree) to five (strongly agree). The total score is the average of all items, with higher scores indicating increased intention to migrate. The original Cronbach alpha value of the scale was 0.91, whereas in this study, it is 0.95. All statistical findings that emerged as a result of CFA supported (Meydan & Şeşen, 2015) the overall measurement quality: CMIN, 2.598; DF, 106; CMIN/DF, 2.598; GFI, 0.927; NF, 0.922; CFI, 0.950; AGFI, 0.863; and RMSEA, 0.075.

#### Psychological well‐being

The Psychological Well‐being Scale (PWBS) was developed by Diener et al. (2009) to measure sociopsychological well‐being as a complement to existing well‐being measures. The scale consists of eight items. The items are answered on a scale of 1−7, ranging from strongly disagree (1) to strongly agree (7). All items are expressed positively. Total scores range from 8 (if strongly disagree with all items) to 56 (if strongly agree with all items). A high score denotes that the person has many psychological resources and strengths. The scale items were constructed by considering the basic components of various well‐being theories. PWBS evaluates critical components of sociopsychological functioning from the individual's perspective. PWBS encompasses various elements, including social relationships, purposeful living, self‐esteem, optimism, and a sense of competence. These constructs involve having supportive relationships, contributing to others’ well‐being, earning respect, finding purpose in daily tasks and work, staying interested and involved, and feeling capable in important endeavors. In cases where these elements are supported, it is hypothesized that PWB will serve as a buffer. Specifically, if the fear of violence heightens the intention to migrate, the presence of these PWB characteristics may lessen the impact. Therefore, PWB was selected as a moderator variable in this context. Telef (2013) investigated the Turkish validity and reliability of the questionnaire. The original Cronbach's alpha value of the scale was 0.87, whereas in this study, it is 0.93. As a result of CFA, it was understood that the PWB measure was suitable for use as in other scales (Meydan & Şeşen, 2015) used in this study: CMIN, 120.338; DF, 33; CMIN/DF, 3.647; GFI, 0.900; NFI, 0.833; CFI, 0.871; AGFI, 0.833; and RMSEA, 0.081.

### Data analysis

Data analysis was performed using SPSS version 22.0 (IBM Corporation, Armonk, NY, USA), AMOS version 22.0 (IBM Corporation, Armonk, NY, USA), and HAYES. AMOS and HAYES (2013), utilized as SPSS add‐on programs, were used for validity, reliability, and structural equation modeling. For the research, regression analysis Model 4 was used. For Model 4, the dependent variable is nurses’ attitudes about emigration, whereas the independent variable is FFVEW, and the moderator variable is PWB. All analyses were performed with a 95% confidence level and a 5% margin of error.

### Ethical statement

Initially, the Süleyman Demirel University Ethics Committee ethically assessed and authorized the research design and methodology (No. E‐87432956‐050.99‐393148). In addition, the researchers fully complied with the guidelines of the Declaration of Helsinki. The purpose and rationale of the study was explained to the participants and both verbal and written agreements were obtained. To ensure anonymity, the questionnaires were collected in closed envelopes.

## RESULTS

### Participant demographics

A total of 221 nurses from two hospitals participated in this poll. In the first hospital, 73.8% of the participants were women, 85.6% were married, and 87% were with undergraduate education. In the second hospital, 94.7% were women, 78.4% were married, and 80.1% had undergraduate degrees. Overall, 87.3% of the participants were women, 81.4% were married, and 83% had undergraduate education. The average age was 36.27 ±8.6 years, and the average work experience was 15.06 ± 10.01 years.

### Descriptive statistics and correlation analysis

Although ASBD (3.31 ± 0.96) and PWB (3.65 ± 0.83) had a medium–high mean for the variables, the mean of FFVEW (2.52 ± 0.97) is lower. According to the Likert scale, though the mean of the participants’ ASBD was above the median value, they agreed more with the comments made concerning migration. This is higher for PWB. They responded to the PWB assertions as agreed on average. However, for FFVEW, they disagreed or completely disagreed with many statements. Based on the average of the participants, it can be concluded that their fear of violence is lower than usual. As a result of the correlation analysis, there was a negative and moderate correlation between PWB and FFVEW (*r* = −0.401, *p* < 0.001), and there was a negative but weak correlation between PWB and ASBD (*r* = −0.247, *p* < 0.001). Also, FFVEW was positively but weakly correlated with ASBD (*r* = 0.325, *p* < 0.001) (Table 1).

**TABLE 1:** Correlation analysis and descriptive statistics.

	Mean	SD	1	2	3
Fear of future violent events at work (1)	2.52	0.97	1		
Psychological well‐being (2)	3.65	0.83	−0.401**	1	
Attitude scale for brain drain (3)	3.31	0.96	0.325**	−0.247**	1

*Note*: **Correlation is significant at the 0.01 level.

### Test of mediation effect

**FIGURE 1:**
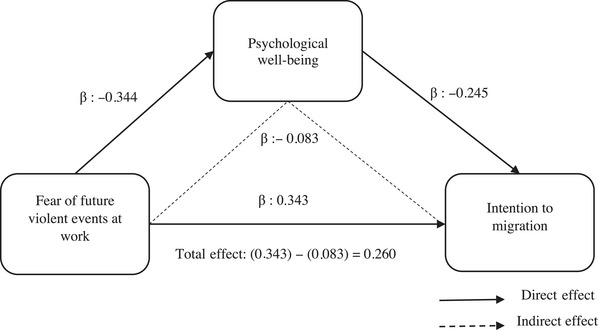
The mediating role of PWB on the effect of FFVEW on intention to migration.

The result of the mediation effect is shown in Table 2. SPSS PROCESS (Model 4; Hayes, 2013) add‐on with bias‐corrected bootstrapping was evaluated using a sample of 10,000 and 95% confidence intervals. The regression weights among FFVEW, ASDB, and PWB were calculated (see Figure 1). According to the model, FFVEW (β = 0.343; *p* < 0.001; *t* = 5.087) positively influences ABSD but negatively impacts PWB (β = −0.245; *p* < 0.001; *t* = −6.487). Also, FFVEW affects PWB (β = −0.344; *p* < 0.001; *t* = −3.764) negatively. The predictive effect of FFVEW in explaining PWB was 16% (R2: 0.161), the predictive effect of FFVEW was 12% (R2: 0.121), and the predictive effect of ASBD of PWB was 12% (R2: 0.120).

**TABLE 2:** Direct and indirect effects.

Variables	Direct effect	Indirect effect	Total effect	LLCI	ULCI	t	p
Fear of future violent events at work	0.343	−0.083	0.260	0.1590	0.4268	4.31	0.000
Psychological well‐being	−0.245			−0.3041	0.0088	−1.85	0.000

*Note*: Dependent variable: Attitude scale for brain drain.

## DISCUSSION

To our knowledge, this is the first study to investigate the relationship between fear of future workplace violence and intention to migrate and the mediating role of PWB in this relationship among nurses in Turkey. This research contributes to the literature in five ways. First, significant connections were found between FFVEW, ASBD, and PWB. There is a negative relationship between FFVEW and ASBD and PWB and ASBD and a positive relationship between FFVEW and ASBD. Second, our findings demonstrated that fear of future violence at work caused an increase in the intention of the nurses to migrate and PWB of nurses reduces the intention to migrate. In the current study, the level of fear of future violence at work was found to be lower compared with studies conducted in China (Fu, Ren, et al., 2021; Fu, Wang, et al., 2021). A study conducted in Portugal (Pacheco et al., 2022) reported similar results regarding nurses’ fear of experiencing workplace violence. As witnessing and experiencing violence increase the fear of being exposed to violence at work in the future (Akbolat et al., 2021; Fu et al., 2023), this fear can be explained by the frequency of violence incidents healthcare workers face institutions worldwide. The fact that prevalence of workplace violence among nurses in China is higher than in Turkey (Çam & Ustuner Top, 2022) and that the prevalence of workplace violence among healthcare workers in Asia is higher than in Europe (Liu et al., 2019a) supports our inference.

Third, our study demonstrated that nurses' mean intention to migrate was moderately high. This result parallels the recent research conducted by the Turkish Nurses Association (2023). Out of the 8,274 participants, 6,310 expressed an intention to migrate and work as a nurse abroad. Among the nurses who expressed an intention to migrate, 25.8% stated psychological and physical violence as the primary factors for their the intention to migrate. In our study, as we expected (hypothesis 1), the factor positively affecting the intention to migrate is the FFVEW. Another study found that workplace violence increases the propensity to migrate among nurses (Boafo, 2016). If we interpret this situation from the perspective of Lee's (1966) push–pull theory, we can state that the fear of experiencing workplace violence is a push factor. Consequently, the more nurses have a fear of future violent incidents at work, the stronger their intention to migrate will be.

Fourth, as expected, we also found that fear of future violence at work negatively affects nurses’ PWB (hypothesis 2). This result is consistent with other research that emphasizes the connection between workplace violence and the PWB of nurses (Fu et al., 2023; Mikkola et al., 2017; Pacheco et al., 2016). Pacheco et al. (2016) found that the fear of experiencing workplace violence negatively affects employees psychologically. Furthermore, Fu et al. (2023) discovered that nurses who have not been exposed to violence but have a strong fear of future violence at work were associated with a greater degree of depressive symptoms. Nurses’ PWB is negatively affected when they are exposed to violence (Hall et al., 2018; Pariona‐Cabrera et al., 2020). However, nurses’ psychological health is negatively impacted due to the fear they endure, even if they do not encounter violence at work. It is known that the psychological negative effects resulting from fear of future violence at work can last for several days to several weeks or even longer (Mikkola et al., 2017). Consequently, nurses’ psychological health is adversely impacted by the fear of experiencing workplace violence, regardless of whether they are exposed to it or not.

Finally, our results demonstrated that a basic level of PWB negatively affects the intention to migrate and serves as a mediating variable in the relationship between the fear of future violence at work and the intention to migrate (hypothesis 3). As the levels of PWB increase among nurses who have an increased intention to migrate due to the fear of future workplace violence, a decrease in their intention to migrate is observed. Migration theories provide diverse viewpoints on why individuals migrate. In this study, we emphasize that nurses’ fear of violence and their PWB should be recognized as significant factors influencing their decision to migrate.

Earlier research on nurses examined the variable of PWB as a mediating factor in the context of correlations between several variables like fear of COVID‐19, work quality of life, and organizational support (Maslakçı et al., 2021; Pahlevan Sharif et al., 2018; Tehranchi et al., 2022). However, to the best of our knowledge, the mediating effect of PWB on the relationship between the fear of experiencing workplace violence and the intention to migrate has not been tested before. This circumstance highlights the initial significance of the ongoing study.

Although our research findings contribute to the literature, important research questions remain unexplored. First, our research was done in Turkey. Researchers may find it beneficial to investigate the phenomenon of workplace violence in healthcare systems across different regions, where its prevalence may vary. This comparative analysis may provide insight into how contextual elements influence healthcare professionals' perspectives. Second, our research focused on nurses’ intentions to migrate rather than their actual migration behavior. In future research, it would be beneficial to delve into the process by which nurses with intentions to migrate either follow through with their plans or choose not to migrate. Comprehensive research on this topic could provide deeper insights into the factors influencing nurses’ migration decisions.

### Limitations

Although the results corroborate the theories, there are certain restrictions of the study. The inability to reach all nurses working in public hospitals limits the generalizability of the study. The study only includes nurses working in hospitals in the city center, therefore, it does not represent the opinions of nurses employed in rural hospitals.

## CONCLUSIONS

This study provides new evidence about the effect of the fear of violence of nurses working in Turkey on their intention to migrate and the mediating role of PWB in this effect. The fear of violence caused an increase in the intention of the nurses to migrate. In addition, the fear of violence negatively affects PWB. It would be good to create macro‐ and micro‐policies to allay the nurses’ fear of being exposed to violence. Moreover, the PWB of nurses reduces the intention to migrate and affects the relationship between fear of violence and intention to migrate. Monitoring the psychological health of nurses and implementing preventive measures will be beneficial in reducing their intention to migrate. In short, reducing nurses’ fear of being exposed to violence and increasing their PWB can help prevent potential nurse migration in Turkey. Various factors that influence both temporary and permanent migration, causing nurse shortage and brain drain, should be identified, and pertinent research should be done. It should also identify what drives the opportunities that are so attractive to nurses and retaliate against opposing push factors. Nurses should be motivated to continue to work. There is a need for a better understanding of the interaction between the factors influencing policymakers to make illuminating decisions on retaining employees and improving the overall health system.

### Implications for nursing and health policy

This study reveals the circumstances of nurses who have encountered, seen, or feared violence. Situations that develop after the event can leave permanent scars. Because of this, not only those who are exposed to violence but also those who experience fear should be identified, and appropriate precautions should be taken. A safer organizational climate and increasing occupational health and safety measures should be prioritized for this. The migration or intention to migrate nurses, vital to the continuous and sustainable health system provision, can cause major issues for the nation concerned. Therefore, health policymakers should be aware of the reasons why nurses migrate or consider migration.

Nurses’ fear of violence is among the key factors influencing their migration to other countries. Although this situation seems optimistic for the recipient countries, it is very worrying for the health status of the source countries. Every precaution to be taken against violence and fear of violence can reduce the intention and conduct of nurses to migrate. Macropolitical measures, including the implementation of harsh penalties such as the deprivation of health services and imprisonment, for individuals who commit or attempt violence against health workers, can be effectively employed. Furthermore, microregulations (such as boosting the number of active security personnel in the field and working effectively) can reduce nurses’ fear of violence.

Finally, one of the most critical factors here is PWB. If nurses’ PWB is high, they will cope with fears of violence more successfully and reduce their intention to migrate. Nurse managers and politicians must follow regulations assessing nurses' PWB in this case. To effectively address this goal, it is essential to conduct periodic measurements using questionnaires to assess the PWB levels of nurses. In addition, one‐on‐one, in‐depth interviews should be conducted with nurses to understand their experiences and struggles better. Based on the collected data, it is possible to identify the challenges faced by nurses with low levels of PWB and take targeted actions to address these issues.

As the measures outlined herein cannot be generalized, it is advised that local governments give particular attention to this issue and identify initiatives that will support PWB. The fact that very few studies in this direction have been published in the literature is significant for future research. It is crucial to begin with research on the individual and organizational elements that influence nurses' psychological health.

## AUTHOR CONTRIBUTIONS

*Conception and design*: Deniz Güneş, Nevin Günaydin, and Mustafa Amarat. *Data collection*: Nevin Günaydin and Mustafa Amarat. *Analysis and interpretation of data*: Mustafa Amarat. *Study supervision*: Deniz Güneş. *Manuscript writing*: Deniz Güneş and Mustafa Amarat. *Critical revisions for important intellectual content*: Deniz Güneş, Nevin Günaydin, and Mustafa Amarat.

## CONFLICT OF INTEREST STATEMENT

The authors declare no conflicts of interest.

## FUNDING INFORMATION

This research received no specific grant from any funding agency in the public, commercial, or not‐for‐profit sectors.

## ETHICS STATEMENTS

Süleyman Demirel University's Ethics Committee (Approval Date: November 16, 2022, Approval Number: E‐87432956‐050.99‐393148) approved the study.
